# Low molecular weight silicones induce cell death in cultured cells

**DOI:** 10.1038/s41598-020-66666-7

**Published:** 2020-06-12

**Authors:** Carla Onnekink, Rita M. Kappel, Wilbert C. Boelens, Ger J. M. Pruijn

**Affiliations:** 1 Department of Biomolecular Chemistry, Institute for Molecules and Materials (IMM), Radboud University, Nijmegen, the Netherlands; 2 Dr. Kappel Institute, Zwolle, The Netherlands

**Keywords:** Biochemistry, Risk factors

## Abstract

Women with silicone gel-filled breast implants are exposed to organosilicon compounds, in particular methylsiloxanes, as a result of ‘gel bleed’ and implant rupture. Although these silicones were originally considered to be inert, increasing evidence indicates that they can cause serious health problems. Here, we have analyzed the effects of microdroplets of the methylcyclosiloxanes, in particular D4, on the viability of cultured human cells. The exposure of Jurkat suspension and HeLa monolayer cells to D4 resulted in morphological changes of the cells. The analysis of molecular markers for apoptotic and necrotic processes not only demonstrated that caspases were activated and DNA was fragmented in Jurkat cells exposed to D4, but that also the permeability of the plasma membrane was altered. The induction of apoptotic pathways by D4 was substantiated by the inhibition of caspase activation in cells overexpressing Bcl-2. Cleavage of the caspase-3 substrate U1-70K appeared to be dependent on the D4 content and the efficiency of cleavage decreased with increasing size of the methylcyclosiloxanes (D4, D5 and D6). In addition to Jurkat cells, D4-induced U1-70K cleavage was also observed in HeLa cells, but not in HEp-2 cells. Taken together, these results indicate that D4 and, to a lesser extent, D5 can activate cell-death-related pathways in a cell type-specific fashion and suggest that this phenomenon may contribute to the development of Breast Implant Illness.

## Introduction

Humans are exposed in a variety of ways to organosiloxane compounds, also called silicones, which are present in many daily used products, such as deodorants, skin and hair care products, drugs and water repellants. Exposures occur from inhalation, ingestion, dermal absorption and surgical implantation. Although silicones were originally believed to be chemically and biologically inert, accumulating evidence indicates that they might have toxic side-effects^1–5^. The implantation of silicone gel-filled breast implants results in a continuous exposure to silicones, because these compounds are known to leak from the implants. The safety of these implants has been debated for a long time^2,4,6^. Especially during the last ten years it has become more generally accepted that the exposure to silicones in some individuals may have adverse health effects^7,8^.

The most common silicone in gel-filled implants is polydimethylsiloxane and its production not only yields polymers, but also cyclic oligomers^9,10^ The cyclic oligomers consist mainly of tetramers and pentamers, which are known as D4 and D5, respectively. Silicone gels that are used in implants are composed of slightly crosslinked chains of polydimethylsiloxanes, so that they form a three-dimensional network. Due to the inherent incompleteness of crosslinking the gels contain 1–2% of methylsiloxanes with very low molecular weights (3–20 siloxane units), with either linear or cyclic structures, including D4, D5 and the hexameric D6^11^. The molecules in the latter are not chemically bound to the crosslinked network, but are retained only by physical means. In a silicone breast implant, the gel network and the free silicone molecules are contained within an elastomeric shell. Hence, there is the possibility for leaking, generally indicated by ‘gel bleed’, of the free silicone molecules and it is likely that there is an inverse correlation between the size of the silicone and the efficiency of bleeding. The cyclic oligomers D4 and D5 will be among the smallest silicones present and thus may be overrepresented in the released population of silicones. The more recently applied prostheses contain a bleed retardation layer, which retards but does not completely abolish silicone bleeding. Moreover, the bleeding silicone polymers behave like softeners and eventually weaken the silicone elastomeric shell, which might enhance the risk of spontaneous ruptures^10^, which for obvious reasons also is associated with the release of silicones.

Silicones released from the implants, either by bleeding or as a result of rupture of the elastomeric shell, can migrate throughout the body as shown by the post mortem analysis of various tissues of a patient who had been exposed to gel bleed from silicone breast implants for 17 years. This patient showed accumulations of silicon-containing compounds in plaques or droplets in several organs and nervous tissue^12^. Silicones are highly lipophilic and, therefore, readily adhere to fatty acids and phospholipids and likely have a tendency to accumulate in adipocytes^13,14^. It is also important to note that silicone breast implants undergo changes in gel properties over time^15^. This may at least in part be caused by bacteria – normal breast tissue is known to be not sterile – that convert polydimethylsiloxanes into various silicon-containing molecules^16,17^. The degradation of silicones may occur inside or close to the implant, but may also occur at distant sites after migration through the body. The resulting silicon-containing products can, at least in part, be less hydrophobic and this may increase the rate by which they are distributed through the body.

Hardly anything is known about the effect of silicone accumulations on the viability of cells. In this study, we have exposed cultured human cells to microdroplets of low molecular weight silicones and investigated the effects on cell viability and the appearance of cell death markers.

## Results

### D4 effects on the morphology of Jurkat cells

**Figure 1:**
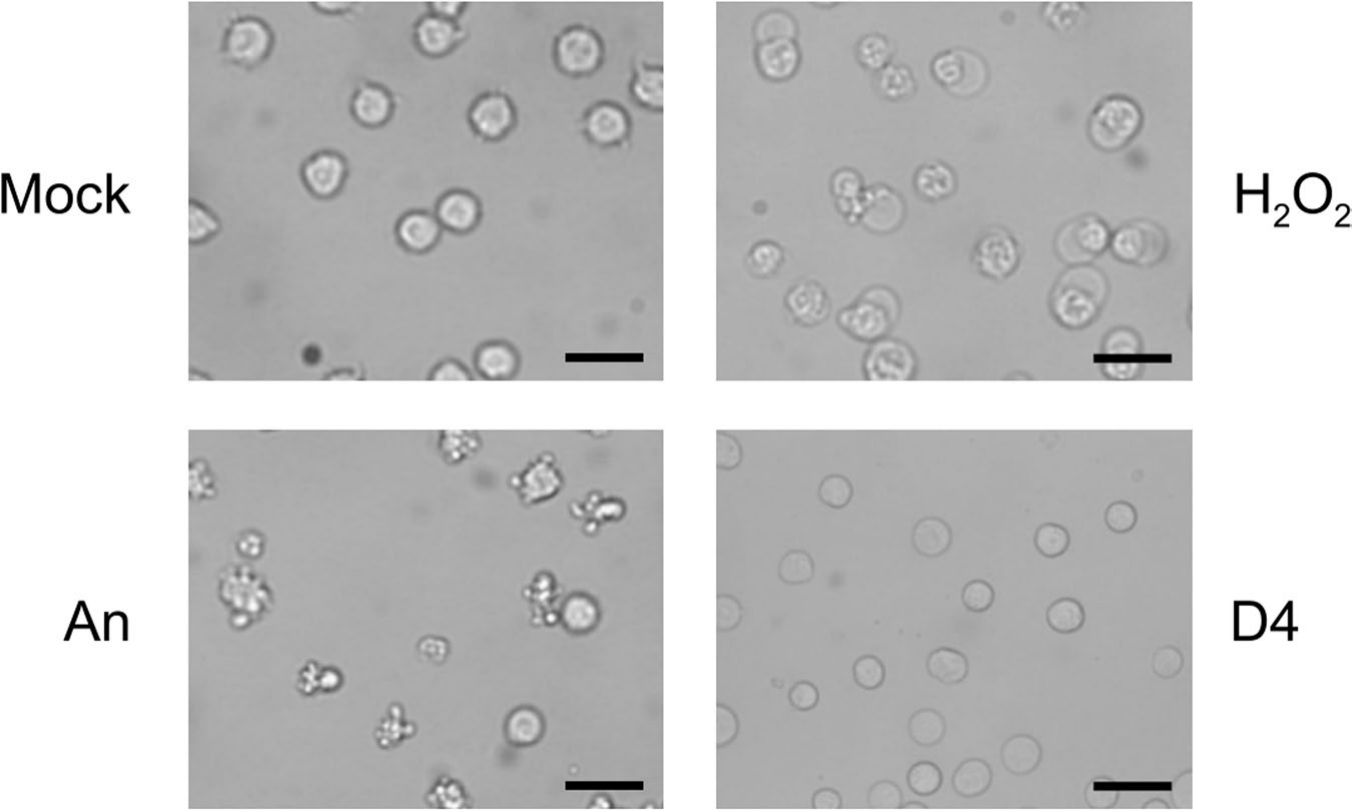
Effects of D4 on Jurkat cell morphology. Jurkat cells, grown in suspension, were cultured for 2 hours in the presence of medium (Mock), 10 μg/ml anisomycin (An), 0.15% H_2_O_2_, or 1% D4. Images were made by phase-contrast microscopy. Bar represents 20 μm.

We chose to investigate the effect of octamethylcyclotetrasiloxane (D4) on the viability of Jurkat cells, which is a human T lymphoblast non-adhering cell line, since D4 is the smallest relatively abundant silicone and probably most able to leak out of implants. D4, like other low molecular weight silicones is a highly hydrophobic oil, and therefore poorly soluble in aqueous solutions (solubility in water at 23 °C: 56 ± 3 ppb). To obtain a mixture, the oil was dispersed in culture medium (at a 1:10 ratio) by sonication. This resulted in small droplets of D4, with an average diameter of approximately 1 μm, in the medium. This emulsion was added to the medium of cells leading to a final D4:medium ratio of 1:100. For at least 8 hours the emulsion was not detectably altered. Their presence in the circulation implies that T lymphocytes may be readily exposed to silicone microdroplets released from implants and the non-adhering behavior of Jurkat cells allows their homogeneous exposure to silicones dispersed in the culture medium. As a reference for treatments affecting cell viability, the cells were treated in parallel with (*i*) anisomycin to induce apoptosis, or (*ii*) H_2_O_2_ to induce necrosis. The induction of these forms of cell death in Jurkat cells has been well documented^18–20^. At several time points after addition of the compounds (2–6 hours), the cells were inspected by bright field microscopy. The exposure of Jurkat cells to D4 in this timeframe did not lead to the characteristic morphological changes of apoptosis or necrosis, such as cell blebbing and cell swelling, which can be observed already 2 hours after the addition of anisomycin and H_2_O_2_, respectively (Fig. 1). However, 2 hours incubation with D4 resulted in a slightly altered appearance of the Jurkat cells, although the overall morphology and size did not or hardly change, suggesting that the physiological state of the cells was affected (Fig. 1).

### Activation of cell death pathways upon D4 exposure

**Figure 2:**
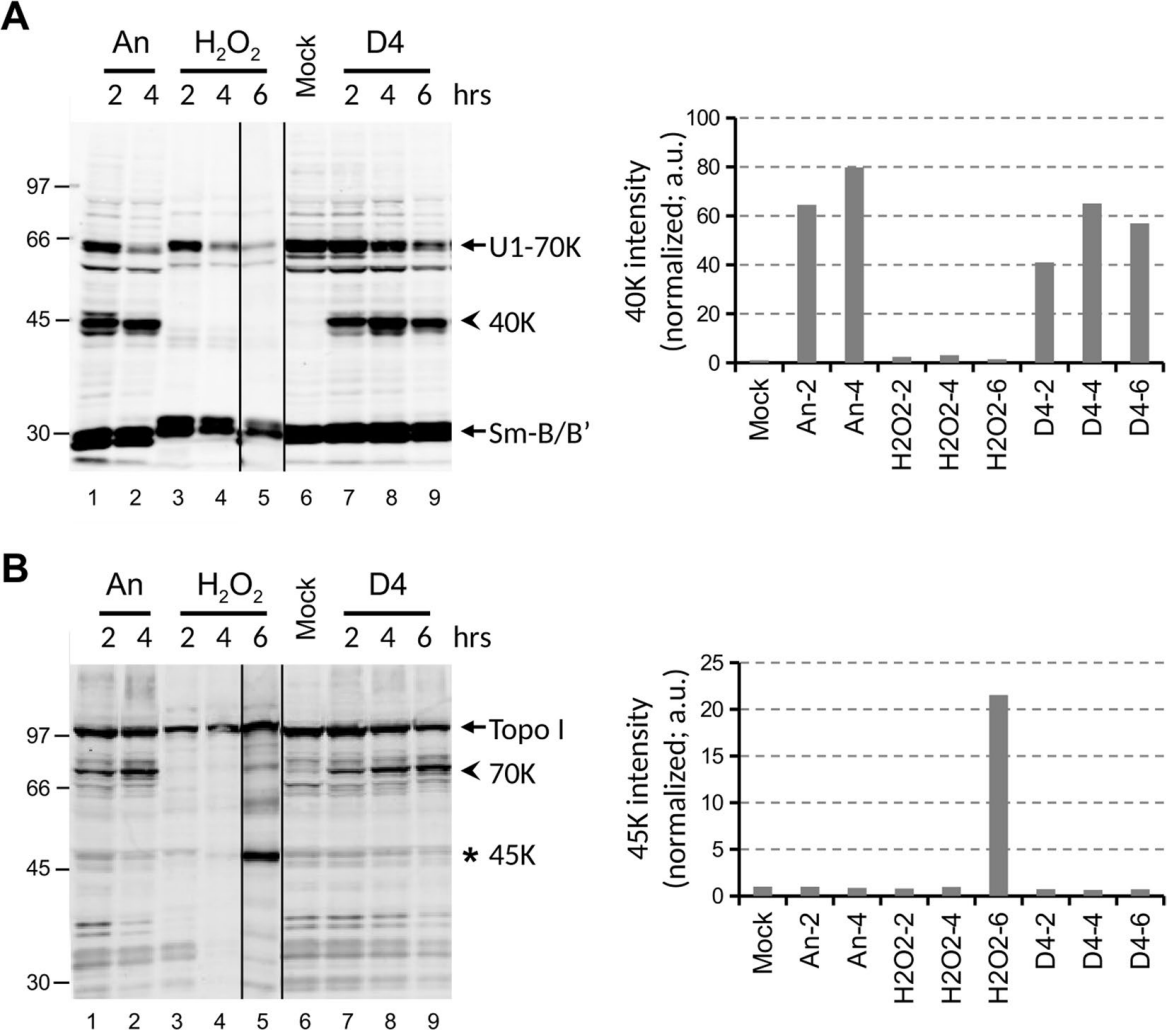
D4 induces the appearance of apoptotic cell death markers in Jurkat cells. Jurkat cells were cultured for the indicated time periods in the presence of 10 μg/ml anisomycin (An), 0.15% H_2_O_2_, or 1% D4. Cell lysates were analyzed by western blotting using patient sera reactive with (**A**) U1-70K or (**B**) Topo I. Note that the anti-U1-70K serum is also reactive with the Sm-B/B’ proteins. The apoptotic cleavage products of U1-70K and Topo I are indicated with arrowheads (40 K and 70 K, respectively); the necrotic cleavage product of Topo I is indicated with an asterisk (45 K). The positions of molecular weight markers are indicated on the left. Note that both panels were composed with different (cropped) parts of the same blot, indicated by the dividing lines, as further illustrated in the Supplementary Information. The intensity of the major cleavage products of U1-70K (40 K) and of Topo I (45 K) was quantified and normalized based upon the Sm-B/B’ signals. a.u.: arbitrary units.

To investigate the effects of D4 on cell viability further, we used biomolecular markers that are specific for apoptosis and necrosis. Lysates of Jurkat cells treated with D4, anisomycin or H_2_O_2_ for various time periods were subjected to immunoblotting with antibodies directed to U1–70K and topoisomerase I (Topo I). The U1-70K protein is cleaved by caspase-3 in apoptotic cells, resulting in a characteristic 40 K fragment^18,19^. Topo I is converted into a 45 K fragment in necrotic cells and into a 70 K fragment in apoptotic cells^20^. As expected, the characteristic cleavage products of U1-70K and Topo I were observed in the Jurkat cells treated with anisomycin or H_2_O_2_ (Fig. 2, panels A and B respectively).Interestingly, also the exposure to D4 led to the appearance of the molecular markers for apoptosis. Both the 40 K fragment of U1-70K and the 70 K fragment of Topo I were observed already 2 hours after the addition of D4 (Fig. 2A,B). These results strongly suggest that caspases are activated in D4-treated Jurkat cells.

**Figure 3:**
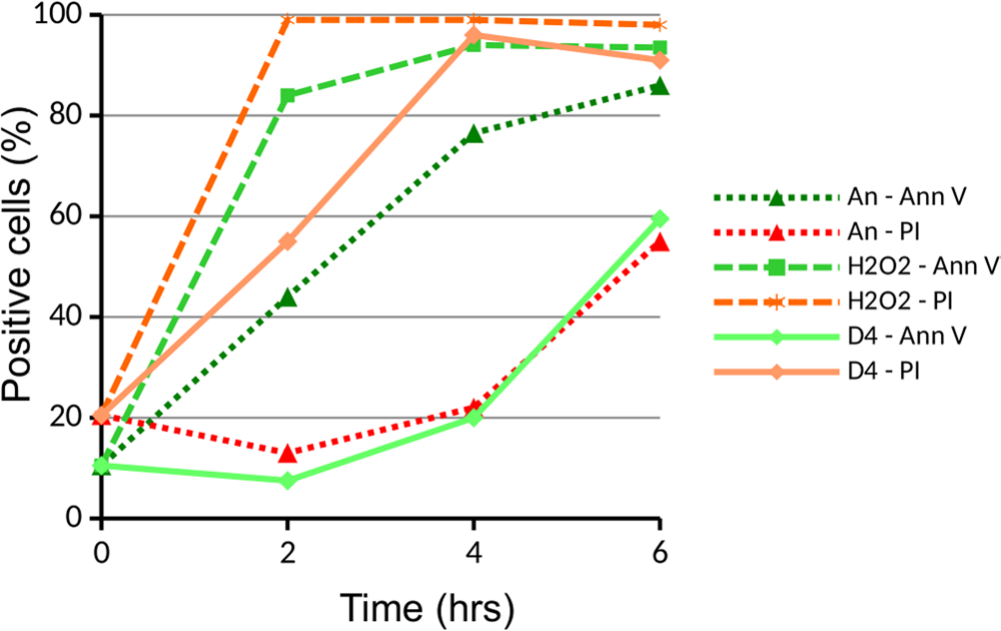
Cell membrane (permeability) changes induced by D4. Jurkat cells were cultured in the presence of 10 μg/ml anisomycin (An), 0.15% H_2_O_2_, or 1% D4. After several time periods the cells were briefly incubated with annexin V-FITC (Ann V) and propidium iodide (PI) and cell staining was monitored by flow cytometry.

To determine whether D4 treatment of Jurkat cells indeed induces apoptosis, additional features associated with apoptosis were investigated. First, the cells were analyzed for the presence of phosphatidylserine at the outer leaflet of the plasma membrane. The cells were stained with fluorescently labeled annexin V (Ann V), a protein that binds to phosphatidylserine, and were analysed by flow cytometry. This analysis was performed in conjunction with propidium iodide (PI), a fluorescent molecule which binds to nucleic acids and stains cells of which the plasma membrane is permeabilized. During the first hours after induction of apoptosis by anisomycin, increased binding of Ann V was observed in Jurkat cells, whereas elevated levels of PI staining, probably as a result of secondary necrosis, were only observed after 6 hours (Fig. 3). In the H_2_O_2_-treated cells, most of the cells were stained with PI and Ann V already after 2 hours, consistent with the necrotic phenotype. The exposure to D4, however, resulted in a relatively rapid increase in PI staining, with almost 100% of the cells stained after 4 hours, whereas Ann V staining only increased after 6 hours (Fig. 3). These results indicate that D4-treatment does not induce the translocation of phosphatidylserine to the outer leaflet of the plasma membrane, like in apoptotic cells. On the other hand, D4 does lead to enhanced uptake of PI by the cells, which does not seem to be due to severe damage to the plasma membrane, because Ann V is not able to enter the cells.

**Figure 4:**
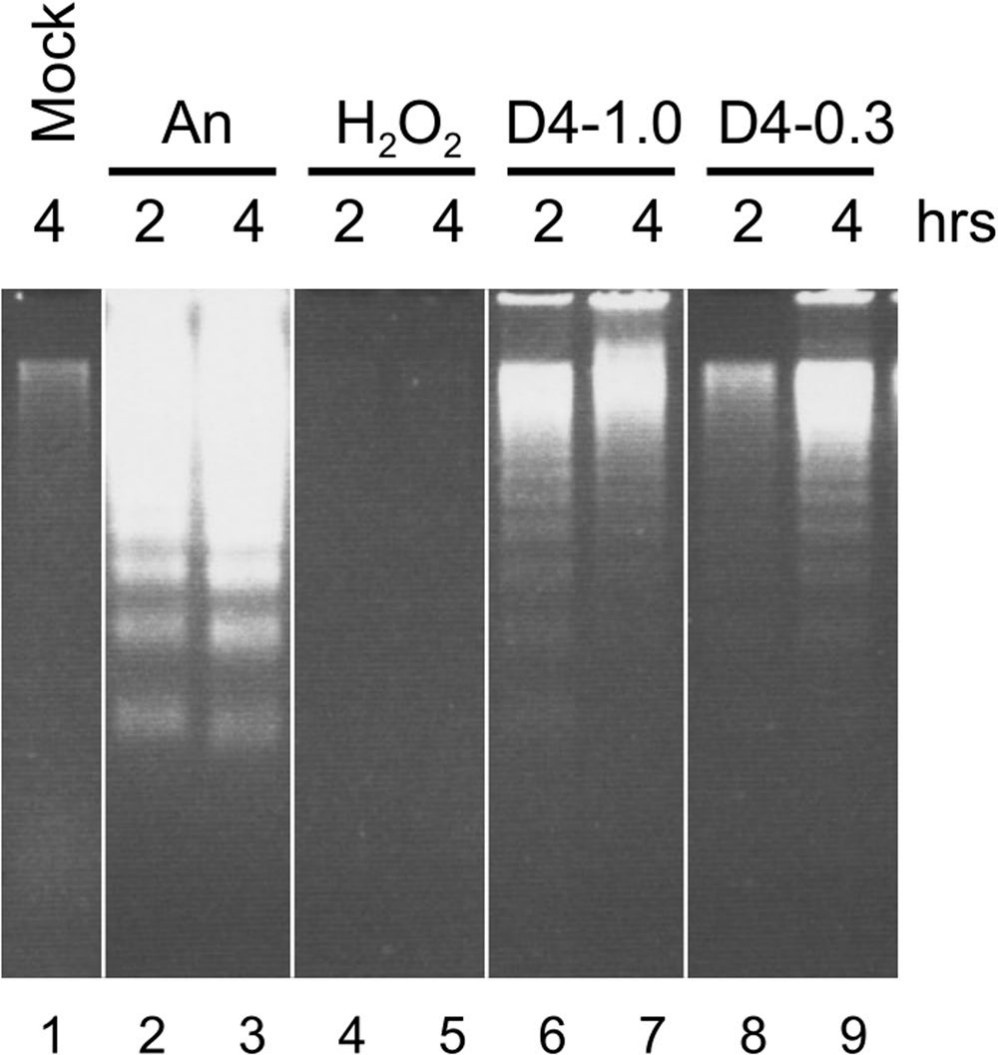
DNA fragmentation in cells exposed to D4. Jurkat cells were cultured for 2 or 4 hours in the presence of 10 μg/ml anisomycin (An), 0.15% H_2_O_2_, 1% D4 or 0.3% D4. Cell lysates were depleted of high-molecular-weight DNA-containing complexes and relatively small fragmented DNA was fractionated by electrophoresis in a 1.5% agarose gel. Note that this figure was composed with different (cropped) parts of the same gel, indicated by the dividing lines, as further illustrated in the Supplementary Information.

Next, genomic DNA was extracted from D4-treated cells and, after depletion of high-molecular-weight DNA-containing complexes, analysed by agarose gel electrophoresis to determine whether D4 induces DNA fragmentation that is typically observed for apoptotic cells. Similar to anisomycin-treated cells, the DNA from D4-treated cells showed the apoptosis-specific ‘DNA laddering’ pattern, albeit less pronounced than what was observed after anisomycin treatment (Fig. 4).

Taken together, the exposure of Jurkat cells to D4 induced several apoptotic processes, such as cleavage of caspase substrates and DNA fragmentation, but apoptotic-like changes at the plasma membrane, such as phosphatidylserine translocation and membrane blebbing were not observed.

### Effects of Bcl-2 on D4-induced caspase activation

**Figure 5:**
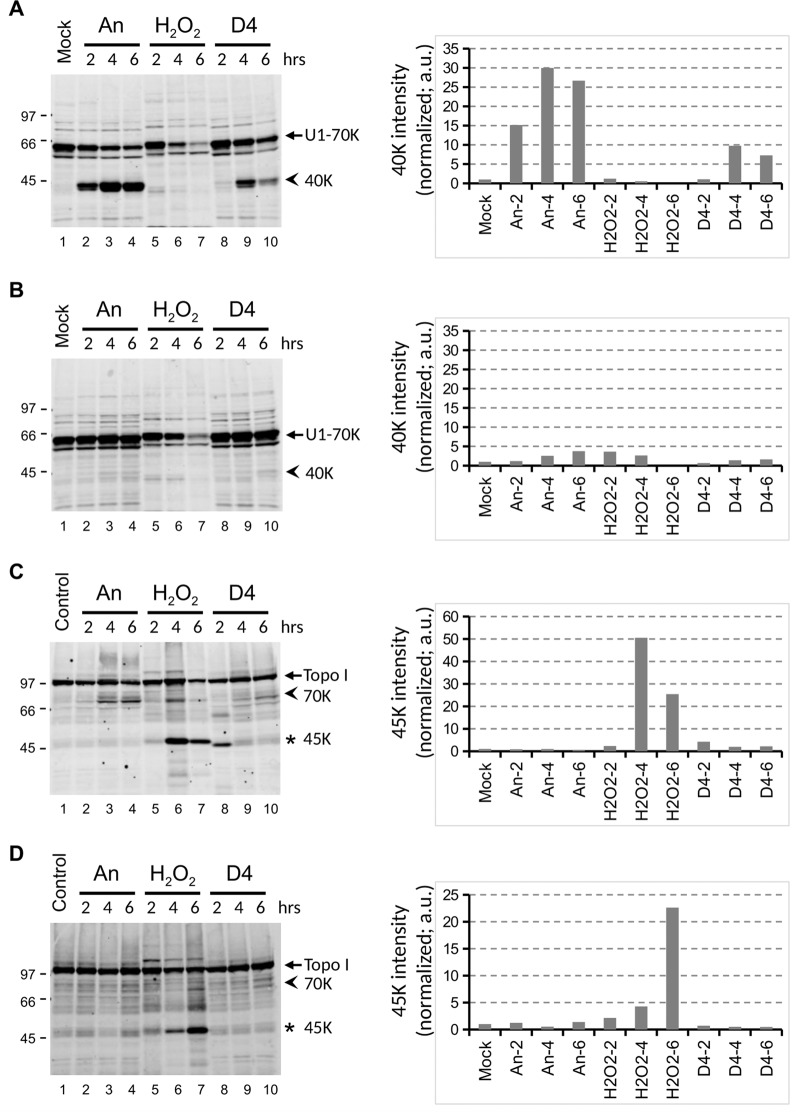
Bcl-2 inhibits cell death induced by D4. Jurkat cells overexpressing Bcl-2 (**B**, **D**) and the corresponding reference cells (Jurkat/Neo) (**A**, **C**) were cultured for the indicated time periods in the presence of 10 μg/ml anisomycin (An), 0.15% H_2_O_2_, or 1% D4. Cell lysates were analyzed by western blotting using patient sera reactive with U1-70K (**A**, **B**) or Topo I (**C**, **D**). The apoptotic cleavage products of U1-70K and Topo I are indicated with arrowheads (40 K and 70 K, respectively); the necrotic cleavage product of Topo I is indicated with an asterisk (45 K). The positions of molecular weight markers are indicated on the left of each panel. Note that in all panels a cropped part of the respective blot is shown, as further illustrated in the Supplementary Information. The intensity of the major cleavage products of U1-70K (40 K) and of Topo I (45 K) was quantified and normalized based upon the Sm-B/B’ signals. a.u.: arbitrary units.

To substantiate that the molecular apoptotic features induced by D4 indeed were due to apoptotic events, stably transfected Jurkat cells overexpressing the apoptosis inhibitor Bcl-2 and a transfection vector control cell line^21,22^ were treated with D4, anisomycin or H_2_O_2_. Immunoblotting of cell lysates with anti-U1-70K and anti-Topo I antibodies demonstrated that the D4-induced cleavage of U1-70K and Topo I was strongly reduced in the presence of Bcl-2, very similar to what was observed upon anisomycin treatment (Compare Fig. 5A with 5B and 5 C with 5D). In contrast, the conversion of Topo I into the 45 K product in H_2_O_2_-treated cells was not affected by Bcl-2 expression (Fig. 5C,D). These results are consistent with D4 inducing apoptosis via the intrinsic pathway, by stimulating the clustering of Bax/Bak at the mitochondrial membrane^23^.

### Dose- and methylcyclosiloxane size-dependency of caspase activation

**Figure 6:**
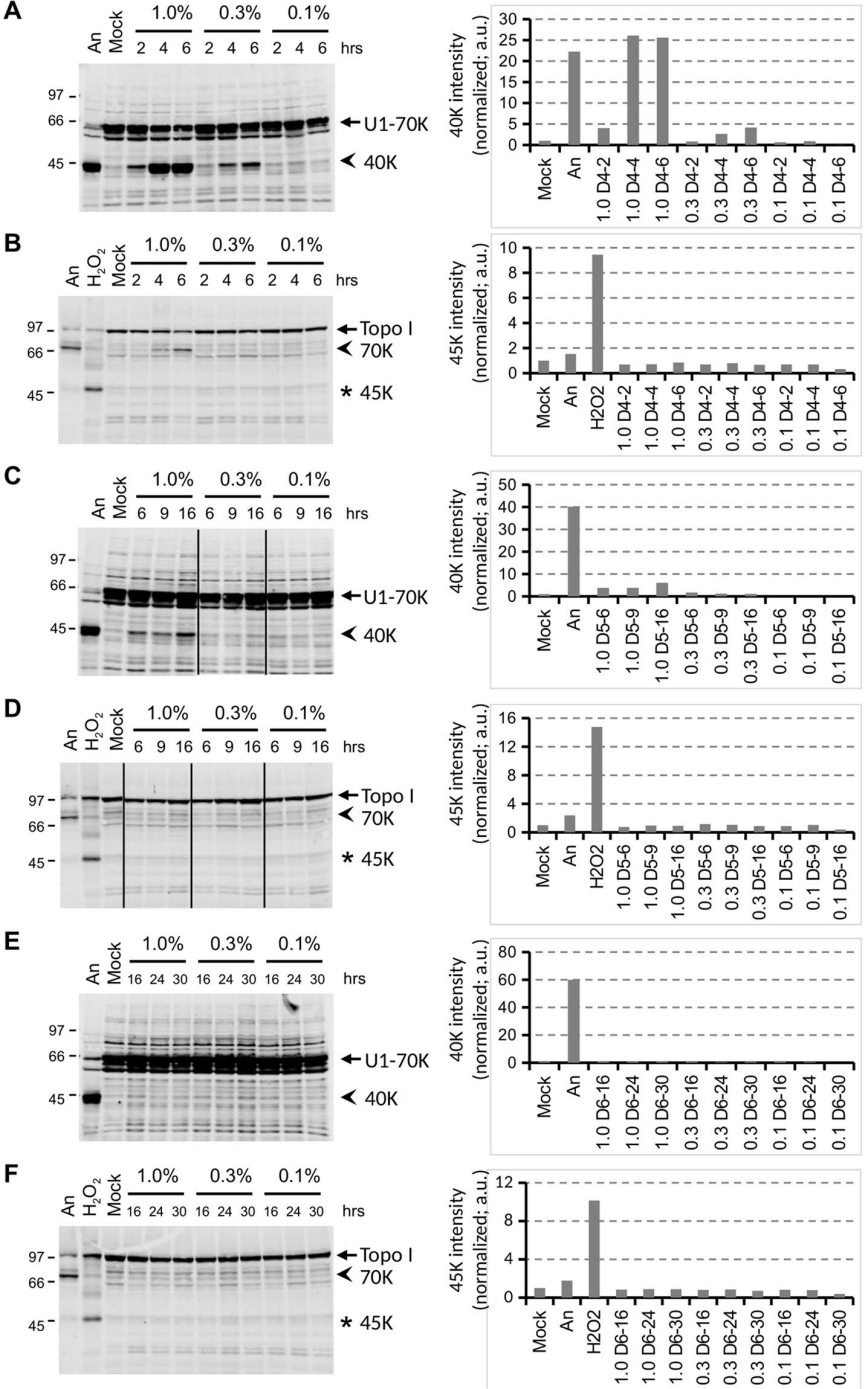
Induction of cell death by methylcyclosiloxanes. Jurkat cells were cultured for the indicated time periods in the presence of various concentrations of D4 (**A**,**B**), D5 (**C**,**D**), or D6 (**E**,**F**). Cell lysates were analyzed by western blotting using patient sera reactive with U1-70K (**A**,**C**,**E**) or Topo I (**B**,**D**,**F**). The apoptotic cleavage products of U1-70K and Topo I (arrows) are indicated with arrowheads; the necrotic cleavage product of Topo I is indicated with an asterisk. As a reference, material from anisomycin-, H_2_O_2_- and mock-treated cells was electrophoresed in parallel (lanes 1-2 in A, C and E; lanes 1-3 in B, D and F). The positions of molecular weight markers are indicated on the left of each panel. Note that in all panels (a) cropped part(s) of the respective blot is shown, if relevant indicated by the dividing lines, as further illustrated in the Supplementary Information. The intensity of the major cleavage products of U1-70K (40 K) and of Topo I (45 K) was quantified and normalized based upon the Sm-B/B’ signals. a.u.: arbitrary units.

To determine to which extent caspase activation was dependent on the dose of D4, Jurkat cells were exposed to emulsions containing 1%, 0.3% or 0.1% D4. Both the apoptotic cleavage of both U1-70K and Topo I decreased with lower quantities of D4 in the culture medium (Fig. 6A,B). At 1% of D4 the U1-70K protein was efficiently converted into the 40 K fragment, whereas the conversion at 0.1% was only very poor.

To determine whether slightly larger methylcyclosiloxanes resulted in similar effects, Jurkat cells were exposed to decamethylcyclopentasiloxane (D5) and dodecamethylcyclohexasiloxane (D6). Immunoblotting analysis of lysates from the treated cells demonstrated that the activation of caspases diminished with increasing molecular weights of the silicones. U1-70K cleavage was still observed after the exposure to 1% D5 (Fig. 6C,D), albeit at lower levels and after longer incubation periods than after D4 treatment, but was not detectable after culturing in the presence of the same dose of D6, even after incubations up to 30 hours (Fig. 6E,F). Similar experiments performed with polydimethylsiloxane microdroplets demonstrated that also under these conditions no cleavage of cell death marker proteins was observed (Supplementary Fig. 1).

These results demonstrate that the induction of the apoptotic events by silicones is dependent both on the amount and the size of the silicone molecules.

### D4-induced caspase activation in other cell lines

**Figure 7:**
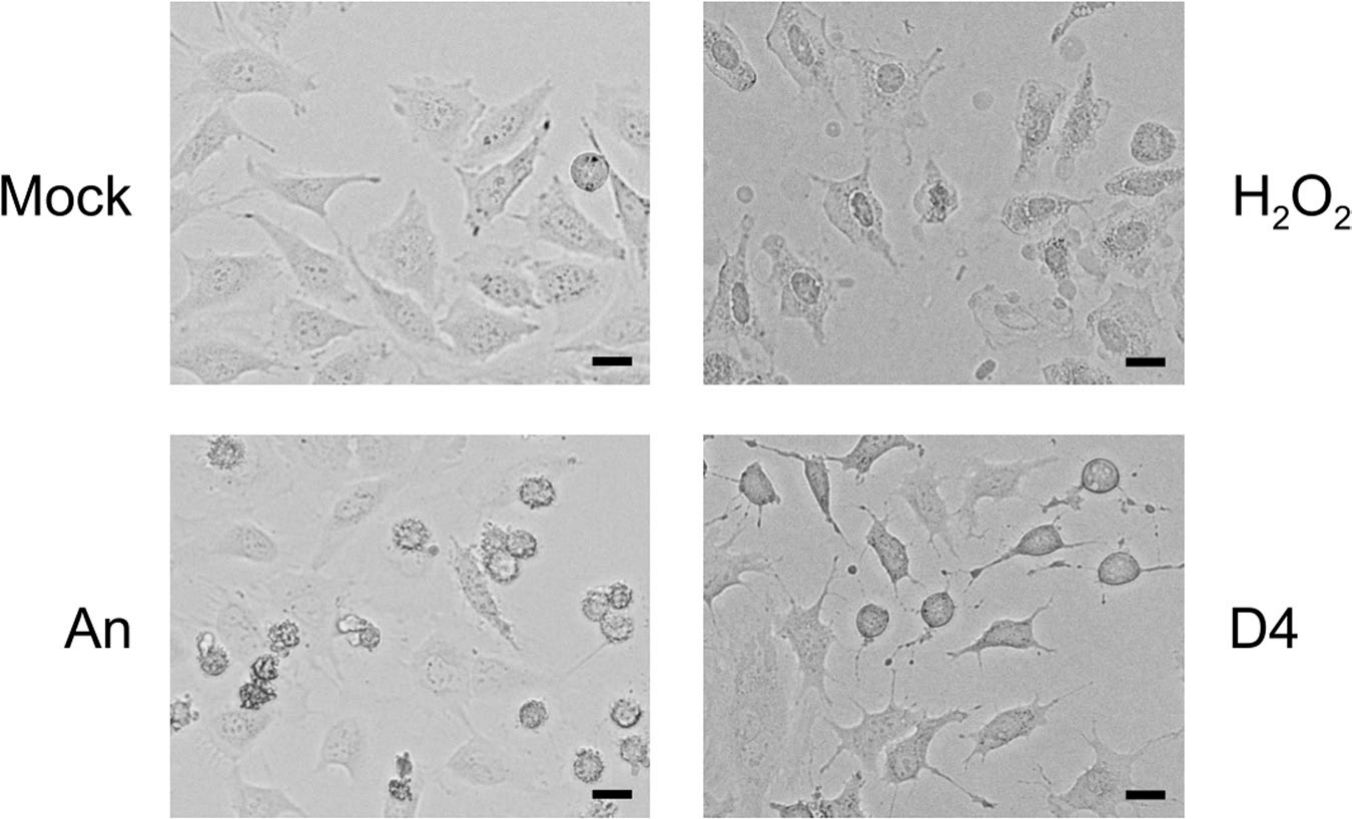
Morphological effects of D4 on HeLa cells. HeLa cells, grown in monolayer cultures, were cultured for 2 hours in the presence of medium (Mock), 10 μg/ml anisomycin (An), 0.15% H_2_O_2_, or 1% D4. Images were made by phase-contrast microscopy. Bar represents 20 μm.

**Figure 8:**
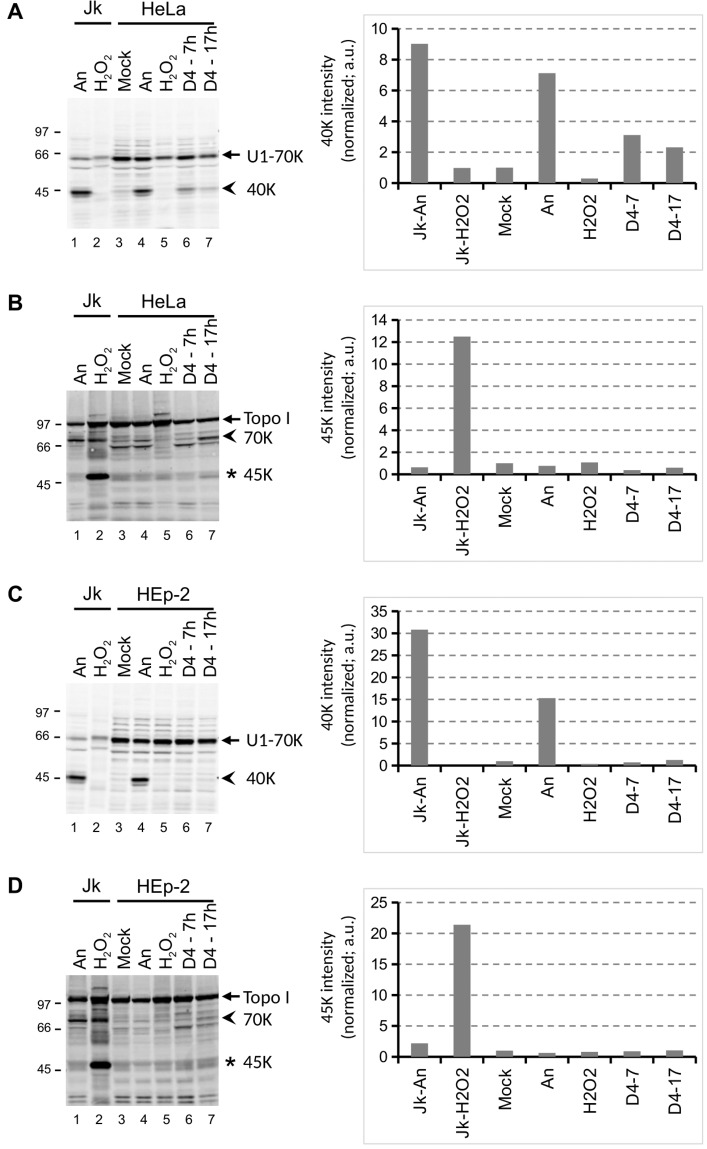
Cell death induction by D4 in HeLa and HEp-2 cell lines. HeLa (**A**, **B**) and HEp-2 (**C**, **D**) cells were cultured for the indicated time periods in the presence of 1% D4, or for 4 hours in the presence of 10 μg/ml anisomycin (An) or 0.15% H_2_O_2_. Cell lysates were analyzed by western blotting using patient sera reactive with U1-70K (**A**,**C**) or Topo I (**B**,**D**). The apoptotic cleavage products of U1-70K and Topo I (arrows) are indicated with arrowheads; the necrotic cleavage product of Topo I is indicated with an asterisk. As a reference, material from anisomycin- and H_2_O_2_-treated Jurkat cells (Jk) was analyzed in parallel (lanes 1-2). The positions of molecular weight markers are indicated on the left of each panel. Note that in all panels a cropped part of the respective blot is shown, as further illustrated in the Supplementary Information.

The sensitivity to toxic compounds might be cell type dependent and to explore this in more detail two other cell lines were treated with D4. The cell types that were used are HeLa, a human epithelial cervix carcinoma cell line and HEp-2, a human epithelial cell line. In agreement with literature data, anisomycin also induced apoptosis in both of these cell lines, as demonstrated by the appearance of the characteristic U1-70K and Topo I cleavage products. The exposure of HeLa cells to D4 resulted in reduced spreading and partial detachment of the cells (Fig. 7). This morphology was clearly distinct from that observed after treatment with anisomycin and H_2_O_2_. Nevertheless, like in Jurkat cells, treatment with D4 resulted in apoptotic U1-70K and Topo I cleavage in HeLa cells, although longer incubation periods were required (Fig. 8A,B). In contrast, the apoptotic cleavage products were not observed in HEp-2 cells incubated with D4 (Fig. 8C,D). Note that the addition of H_2_O_2_ did not lead to the appearance of the Topo I-derived necrosis marker in HeLa and HEp-2 cells.

Taken together, the results of these experiments indicate that the effects of D4 can differ among different cell types.

## Discussion

Although the safety of silicones has been the subject of extensive debate in the past, it is now generally recognized that silicone gel-filled breast implants can cause a disorder, often indicated with Breast Implant Illness. However, hardly anything is known about the molecular and cell biological mechanisms involved in the generation of this disease. Here, we show that the exposure of human cells to low molecular weight methylcyclosiloxanes can induce death of cultured cells. This phenomenon appeared to be dependent on the size of the methylcyclosiloxanes – the effects were most severe for the smallest siloxane (D4) analyzed – and was observed in only two of the three cell types analyzed.

In a recent study, the effects of hexadimethylsiloxane, octamethyltrisiloxane and decamethylcyclopentasiloxane on the viability of differentiated PC12 neuronal cells was studied^24^. Cell viability, which was assessed between 1 and 6 days of exposure, was only affected in the presence of the highest amounts of silicones (silicone:medium ratio 1:5 or 1:10) and was explained by the formation of a silicone film over the culture medium, preventing air exchange. The difference in the experimental setup compared to our study (direct addition versus microdroplets) and the different cell type used impede a direct comparison with our results.

The mechanisms of cell death are based on morphological differences and distinct biochemical signaling and can be categorized in apoptotic and non-apoptotic pathways^25,26^. Distinct characteristics of apoptotic cells are cell shrinkage, nuclear condensation, plasma membrane blebbing, fragmentation of chromosomal DNA, and segregation into apoptotic bodies that are cleared by phagocytosis^27^. Apoptotic processes are initiated by the activation of caspases, a family of proteases that subsequently cleave various cellular proteins^28^. The small methylcyclosiloxane D4 caused cell shrinkage (HeLa cells), chromosomal DNA fragmentation and caspase activation, but did not induce the morphological changes characteristic of apoptosis. Note that cell shrinkage observed in the presence of D4 was different from that observed upon induction of apoptosis with anisomycin.

Necrosis has been regarded as an accidental cell death mechanism in response to environmental perturbations^29^. In contrast to apoptosis, necrosis lacks a key molecular signature and results in uncontrolled activation of cell death. Necrosis is characterized by cellular swelling, plasma membrane damage and uncontrolled release of cell components into the extracellular space. Necrotic cell remnants are cleared by inflammatory cells such as leukocytes and phagocytes^30^. Necrosis can be monitored by the cleavage of Topo I into a 45 K fragment^20^. In our experiments we compared the effects of methylcyclosiloxane with that of hydrogen peroxide, which leads to necrosis as a result of an overload of reactive oxygen species (ROS). The lack of cell swelling, the relatively slow staining of the cells by annexin V and the absence of the 45 K Topo I fragment upon D4 exposure indicate that the effects of D4 do not resemble those of necrosis.

Silicones are very lipophilic molecules and, therefore, are likely to adhere to plasma membrane lipids. When cells are exposed to silicone microdroplets, these silicones may penetrate the lipid bilayer of the plasma membrane, which will result in a dose-dependent disturbance of plasma membrane integrity. Even if the bilayer remains intact, the changed molecular composition of the membrane can affect its fluidity and permeability. The relatively fast uptake of propidium iodide by cells exposed to D4 indeed suggests an altered permeability for lipophilic molecules. In addition, the possibility exists that the altered composition of the membranes affects apoptosis-related changes like blebbing and phosphatidylserine flipping. In necrotic cells the plasma membrane is disrupted early and this is reflected by the early staining of the cells by annexin V, which enters the cells and binds to the phosphatidylserines which are located at the internal leaflet of the plasma membrane. However, it should be noted that phosphatidylserine exposure on the outer leaflet of the plasma membrane has also been observed for regulated non-apoptotic cell death^31^. During apoptosis phosphatidylserines are transferred from the cytoplasmic surface to the cell surface and this explains the staining of apoptotic cells before the plasma membrane is disrupted and propidium iodide can enter the cell (as a result of secondary necrosis). The relatively late staining of D4-treated cells by annexin V not only shows that in these cells phosphatidylserine is not transferred to the outer surface of the cells, but this is also consistent with a relatively late disruption of the plasma membrane.

Overexpression of Bcl-2 prevented the induction of apoptosis-like cell death by D4. Bcl-2 is the founding member of the Bcl-2 family of proteins that dictates cell survival by sequestering activated pro-apoptotic BH3-only proteins and this prevents Bax and Bak to induce apoptosis^23^. In this way Bcl-2 can prevent permeabilization of the outer mitochondrial membrane and the resulting activation of the caspase cascade. The ability of Bcl-2 to inhibit D4-induced apoptosis suggests that D4 activates one or possibly several pro-apoptotic BH3-only proteins resulting in the activation of Bax and Bak. It would be interesting to elucidate which BH3-only protein(s) is/are activated by D4 to understand in more detail what kind of cytotoxic stress is caused by D4. More insight in this pathway may also explain cell-to-cell differences in the response to D4, as cells can have very different levels of BH3-only proteins.

The results in Fig. 6 imply that the efficiency by which caspases are activated in Jurkat cells decreases with increasing size of the methylcyclosiloxane (D4 > D5 > D6). It is currently not known why D4 is a more potent cell death inducer than D5 and D6, but the ease by which these methylcyclosiloxanes penetrate the plasma membrane may be size-dependent.

As already mentioned above, the small methylcyclosiloxanes are known to be among the non-crosslinked low molecular weight silicones of silicone gel-filled breast implants^9,32,33^. Although barrier layers were introduced to reduce gel bleed, these small silicones are expected to be relatively abundant among the silicones that pass the implant’s envelop during gel bleed^11^. Silicone gel-filled implants contain medical grade silicones, in which only low levels of methylcyclosiloxanes are tolerated and, therefore, the remaining small methylcyclosiloxanes are washed out. Nevertheless, relatively high amounts of methylcyclosiloxanes have been found in silicone breast implants in several studies, even in implants that were manufactured recently, and the implants have been found to vary considerably in composition when implants from different brands are compared^33–35^. Silicones released from breast implants are known to migrate through the body and to accumulate at specific locations. As a consequence, it is virtually impossible to give an accurate estimation of the amount of silicones to which cells are exposed at these locations and whether this reflects the amounts that induce cell death in cell cultures. Moreover, as a result of continuous ‘bleeding’ of the silicones and the degradation of silicones (described above) the amount of small silicones at such locations likely increases over time. In this respect it is also important to note that the sensitivity of different cells for silicone toxicity may show strong differences.

Although D4 readily activated cell death pathways in Jurkat and, albeit less efficiently, in HeLa cells, caspase activation was not observed in HEp-2 cells, which indeed suggests that human cells are differentially sensitive to methylcyclosiloxanes. While U1-70K cleavage was induced in lymphoblast and epithelial cervix carcinoma cell lines, the epithelial cell line did not show this phenomenon upon exposure to D4. The reason for this differential sensitivity is currently not known and clarification awaits the elucidation of the molecular mechanisms of cell death induction by methylcyclosiloxanes.

Although silicone gel-filled breast implants were originally proposed to trigger immune-mediated ailments in certain individuals, during the last decade it became clear that they may cause a systemic illness that is not necessarily immune-mediated. This illness, currently often indicated with Breast Implant Illness, is characterized by chronic fatigue, fibromyalgia, dry eyes, mouth and skin, shortness of breath, recurrent infections, several neurological complaints and many other symptoms^36,37^. Nevertheless, the causal relationship between silicone breast implants and a related illness remains a matter of debate^38^.

Silicones passing the shell of the implants during gel bleed migrate to the layer between the implant and the inner surface of the periprosthetic capsule. The released silicones will be emulsified in the periprosthetic fluid and may, subsequently, migrate through the periprosthetic capsule, enter the (lymphatic) vessels and then can be transported to other, distant parts of the body. During or after their distribution many cells and tissues may become exposed to these silicone microdroplets. If these cells are sensitive to methyl(cyclo)siloxanes present in these droplets, cell death pathways may be activated, like in the cultured cells, and this may lead to tissue degeneration, functional impairment, or the activation of the immune system. Each of these phenomena can contribute to the pathophysiology of Breast Implant Illness and the location where this occurs will be related to specific symptoms.

Although cultured cells mimic cells in a certain tissue in the body, they do not fully reflect the properties of cells in their natural environment, not only because they are immortalized, but also because the microenvironment is not identical to the *in vivo* situation. Nevertheless, the composition of the membranes and the biochemical and signaling pathways will be very similar, if not identical, and as a consequence the effects of the exposure to methylcyclosiloxanes will probably be the same. As described above, silicones released from implants are expected to form emulsions in the periprosthetic fluid, which will lead to microdroplets to which the cells will be exposed. To mimic this situation as much as possible, the silicone oils were dispersed in culture medium by sonication. It should, however, be noted that the size and composition of the resulting microdroplets may differ from those generated by gel bleed from implants and, as a consequence, cannot be directly extrapolated to the situation in patients with Breast Implant Illness.

In conclusion, our data show that the small methylcyclosiloxanes D4 and, to a lesser extent, D5 can induce cell death related events in cultured human cell lines in a cell type-specific manner. Although a number of these events are also observed in apoptotic cells, the process induced by the silicones does not completely resemble apoptosis. The results suggest that the release of silicones from breast implants by gel bleed or implant rupture leading to the generation of tiny droplets that migrate through the body may affect health by triggering cell death in certain organs and tissues.

## Methods

### Cell lines

Jurkat (human T cell leukemia) cells were grown in RPMI-1640 medium (Gibco-BRL) supplemented with 10% heat inactivated fetal calf serum (FCS), 1 mM sodium-pyruvate and penicillin (100 U/ml) and streptomycin (100 μg/ml).

Jurkat cells, with Bcl-2 (Jurkat/Bcl-2) or without Bcl-2 (Jurkat/Neo) overexpression (a kind gift of John Reed, La Jolla, CA, USA), were grown in RPMI-1640 (Gibco-BRL) medium supplemented with 10% heat-inactivated fetal calf serum, 200 μg/ml G418 (Gibco-BRL), 1 μM β-mercapthoethanol, 1 mM sodium-pyruvate and penicillin and streptomycin. Jurkat/Neo represents a cell line stably transfected with the transfection vector that was used to generate the Jurkat/Bcl-2, but lacking the Bcl-2 cDNA. These cell lines originate from the same parent cell.

HeLa and HEp-2 cells were grown in DMEM supplemented with Glutamax (Gibco) and 10% FCS, penicillin and streptomycin.

### Induction of cell death

To induce apoptosis cells were seeded at a concentration of 1×10^6^ cells/ml (Jurkat) and incubated with 10 μg/ml anisomycin, or plated and grown till approximately 90% confluency and incubated with 10 μg/ml anisomycin (HeLa, HEp-2).

To induce necrosis cells were incubated with 0.15% H_2_O_2_.

Cells were incubated at 37 °C for the indicated time periods before harvesting. After induction of cell death, cells were washed twice with PBS and used immediately or stored at −20 °C.

### Silicone oils

A volume of 30 μl silicone oil, D4 (Octamethylcyclotetrasiloxane, 98%, Aldrich), D5 (Decamethylcyclopentasiloxane, 97%, Aldrich), or D6 (Dodecamethylcyclohexasiloxane> 98%, TCI Chemicals) was added to 270 μl DMEM without FCS in a 1.5 ml Eppendorf vial, and the silicone oil was dispersed in the medium by 10 min sonication in a Bioruptor (Diagenode) at high setting, 30”/30” interval, 4 °C. To expose cultured cells to the dispersed silicone oil, this emulsion (0.1 vol.) was added to the cells cultured in the same medium leading to a final silicone:medium ratio of 1:100, unless stated otherwise. The emulsion was stable for at least 8 hours.

### Flow cytometry

Induction of apoptosis or necrosis was monitored by staining the cells with annexin V-FITC in binding-buffer (Abcam) for 10 min on ice, followed by washing with binding buffer. Staining was monitored by a FACSCalibur flow cytometer (BD Biosciences). Propidium iodide (5 μg/ml; Abcam) was added to the cells just prior to measurement.

### Preparation of cell extracts and western blot analysis

Cells were lysed on ice in NP-40 lysis buffer (50 mM Tris-HCl, pH 7.6, 100 mM KCl, 1 mM DTT, 1 mM EDTA, 0.1% NP40, containing Complete protease inhibitor cocktail (Roche). Lysates were sonicated in a Bioruptor (Diagenode) for 5 min at 4 °C and centrifuged for 5 min at 4 °C (12,000 g). Supernatants were used immediately or stored at −20 °C.

Sample buffer (250 mM Tris-HCl, pH 6.8, 10% β-mercaptoethanol, 4% SDS, 20% glycerol and 0.06% bromophenolblue) was added (1 vol.) and the lysates were incubated at 95 °C for 5 min. Proteins were separated by electrophoresis in 10% SDS-polyacrylamide gels and transferred to nitrocellulose membranes (Protran).

Western blots were blocked with 5% skimmed milk in PBS, 0.05% Tween-20 and incubated with anti-U1-70K antiserum (patient serum B156, 1:2,500) or anti-topoisomerase I (Topo I) antiserum (patient serum S119 1:2,500). These patient sera were collected in 1989 in accordance with the ethical guidelines and regulations that were in force in the Netherlands in that period. The serum samples were kindly provided by the rheumatologists of the Radboud University Medical Center who were handling these patients. The autoantibody specificities of these sera have been established previously^39^. Subsequently, the blots were incubated with fluorescent secondary antibody IRDye800 CW-conjugated goat-anti-human IgG (1:5,000) (LI-COR Biosciences) and bound antibodies were visualized by a LI-COR Odyssey imager. The production of the major cleavage products of U1-70K (40 K) and of Topo I (45 K) was quantified by normalizing the fluorescent band intensities based upon the Sm-B/B’ band intensities. Relative band intensities were subsequently determined in relation to the signals for mock treated cell lysates.

### DNA fragmentation

Cells were harvested after treatment, washed with PBS and resuspended in Tris-EDTA buffer (10 mM Tris-HCl, pH 8.0, 1 mM EDTA) and incubated on ice for 30 minutes. Cells and cell remnants were centrifuged at 12,000 g for 15 min at 4 °C to separate soluble material (a.o. fragmented DNA) from nuclei and high-molecular-weight DNA-containing complexes. The supernatant was treated with RNase A (50 μg/ml) at 37 °C for 1 h, extracted with phenol/chloroform, precipitated with isopropanol. The remaining DNA was separated by electrophoresis in a 1.5% agarose gel.

## Supplementary information

Supplementary information.

## Data Availability

The datasets used and/or analysed during the current study are available from the corresponding author on reasonable request.
