# Supplementary material for: Low molecular weight silicones induce cell death in cultured cells
Source: Sci Rep. 2020 Jun 12;10:9558. doi: 10.1038/s41598-020-66666-7 (PMC7293294; doi:10.1038/s41598-020-66666-7)

Supplementary Information

Low molecular weight silicones induce cell death in cultured cells

Carla Onnekink[1], Rita M. Kappel[2], Wilbert C. Boelens[1], Ger J.M. Pruijn[1]*

[1]Department of Biomolecular Chemistry, Institute for Molecules and Materials (IMM), Radboud University, Nijmegen; [2]Dr. Kappel Institute, Zwolle, The Netherlands

Supplementary Figure 1

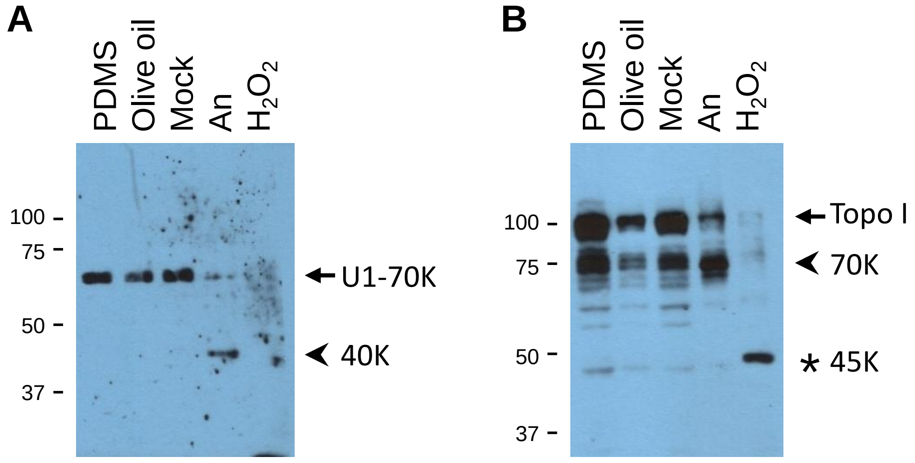

Suppl. Fig. 1 Polydimethylsiloxane does not induce cell death. Jurkat cells were cultured for 7 hours in the presence of polydimethylsiloxane (PDMS) or olive oil. Cell lysates were analyzed by western blotting using patient sera reactive with U1-70K (**A**) or Topo I (**B**). The apoptotic cleavage products of U1-70K and Topo I (arrows) are indicated with arrowheads; the necrotic cleavage product of Topo I is indicated with an asterisk. As a reference, material from anisomycin- (An), H_2O_2- and mock-treated cells was electrophoresed in parallel. The positions of molecular weight markers are indicated on the left of each panel. Note that in both panels a cropped part of the respective blot is shown, as further illustrated below.

Full-length gels and blots

Figure 2

Panel A

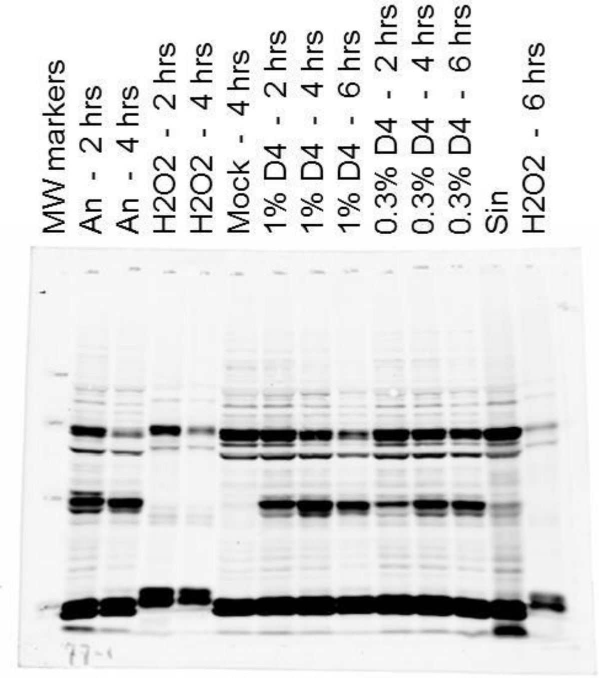

Lane numbers in Figure 2A 1 2 3 4 6 7 8 9 - - - - 5

Panel B

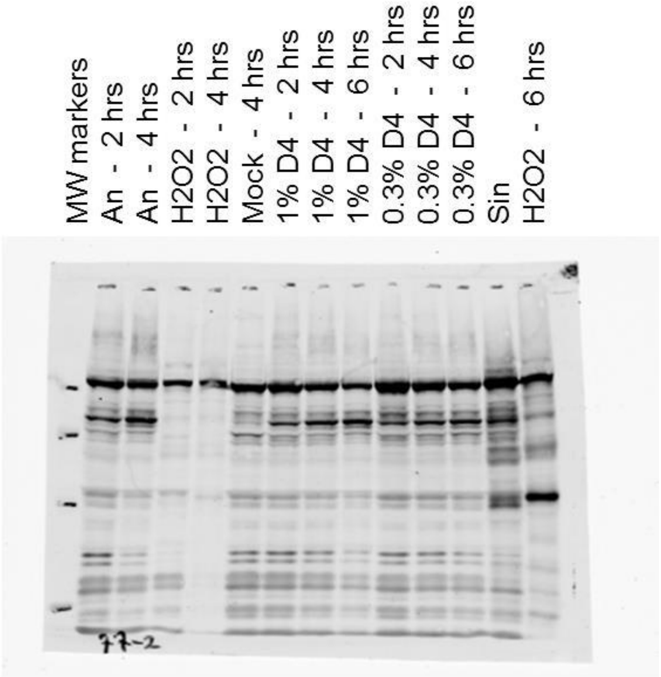

Lane numbers in Figure 2B 1 2 3 4 6 7 8 9 - - - - 5

Figure 4

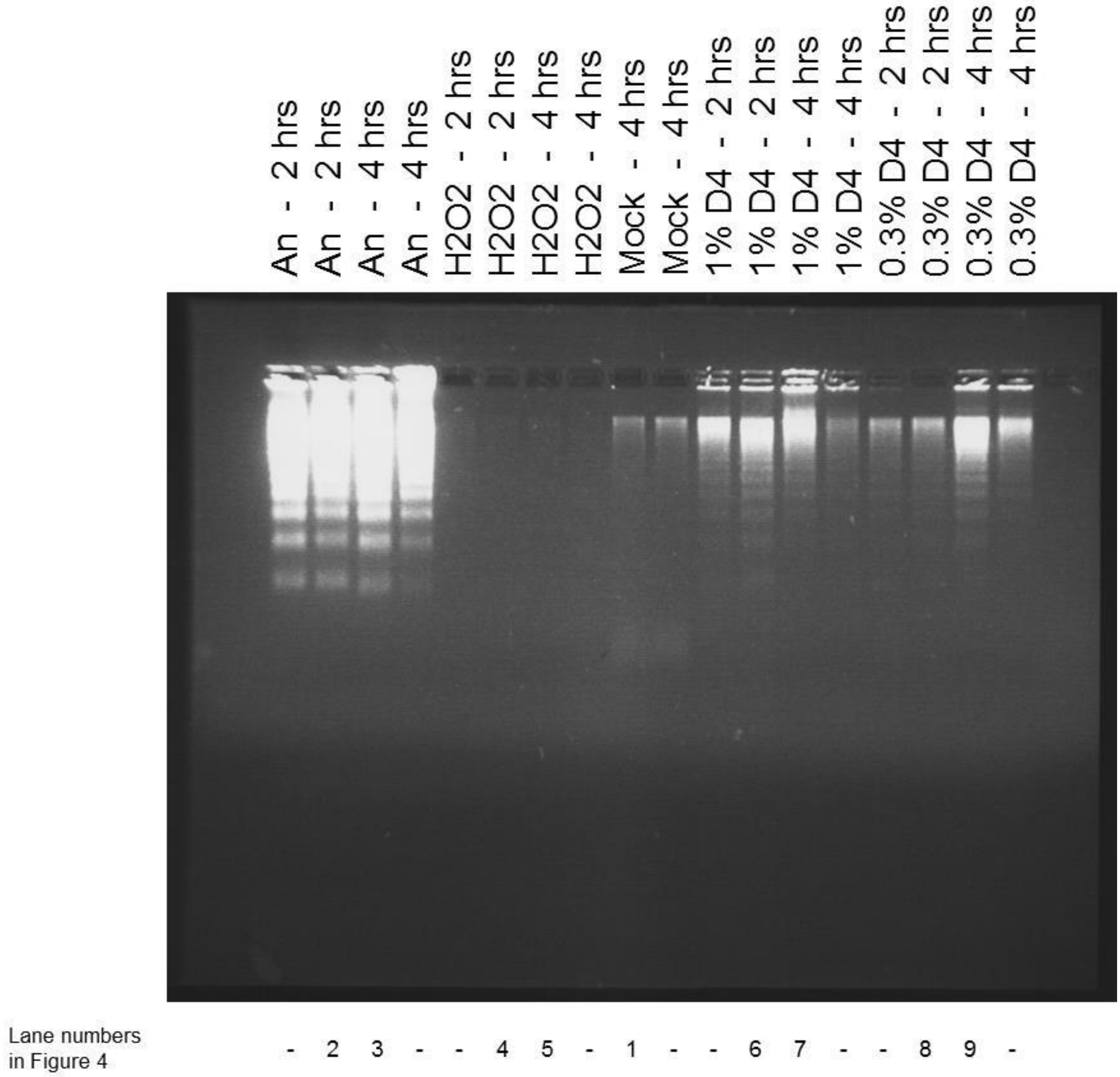
An - 2 hrs
An - 2 hrs
An - 4 hrs
An - 4 hrs
H2O2 - 2 hrs
H2O2 - 2 hrs
H2O2 - 4 hrs
H2O2 - 4 hrs
Mock - 4 hrs
Mock - 4 hrs
1% D4 - 2 hrs
1% D4 - 2 hrs
1% D4 - 4 hrs
1% D4 - 4 hrs
0.3% D4 - 2 hrs
0.3% D4 - 2 hrs
0.3% D4 - 4 hrs
0.3% D4 - 4 hrs
Lane numbers in Figure 4
- 2 3 - - 4 5 - 1 - - 6 7 - - 8 9 -

Figure 5

Section used in Figure 5A marked in red

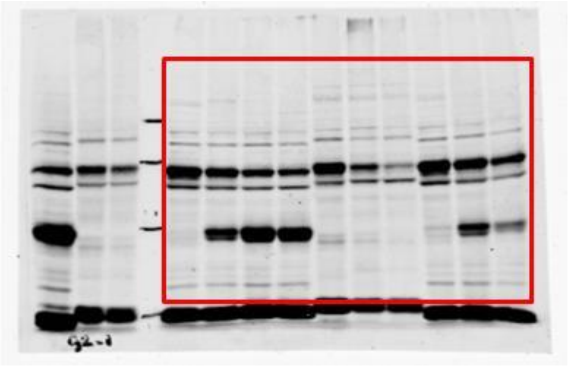

Panel A

Section used in Figure 5B marked in red

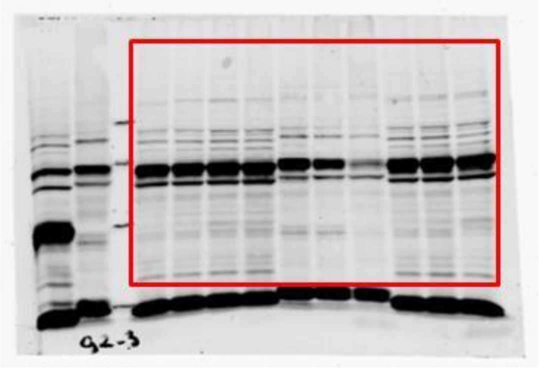

Panel B

Section used in Figure 5C marked in red

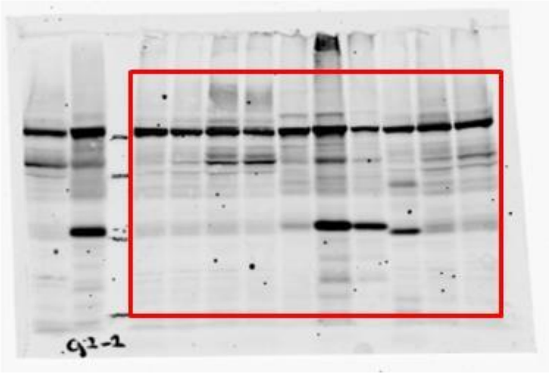

Panel C

Section used in Figure 5D marked in red

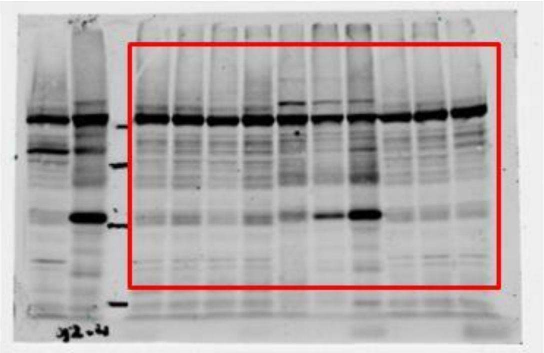

Panel D

Figure 6

Panels A, B

Section used in Figure 6A
marked in red

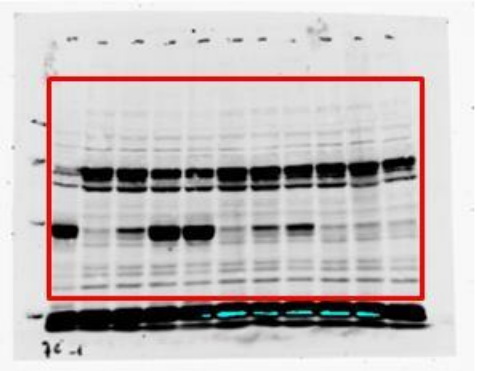

Section used in Figure 6B
marked in red

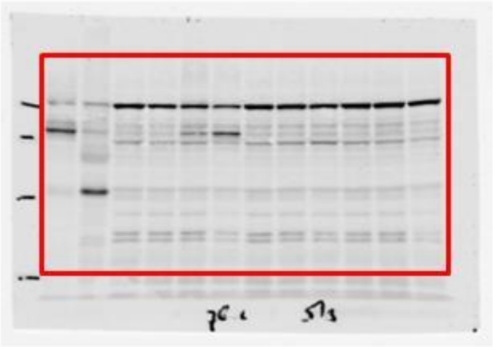

Panels C, D

Sections used in Figure 6C
marked in red

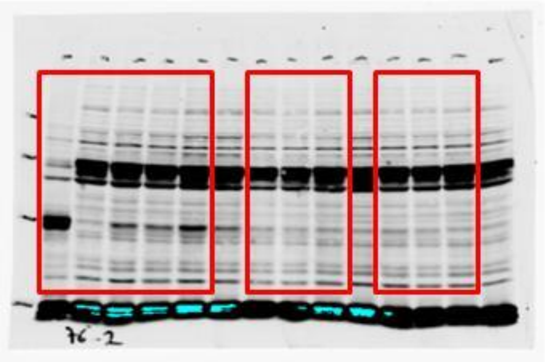

Sections used in Figure 6D
marked in red

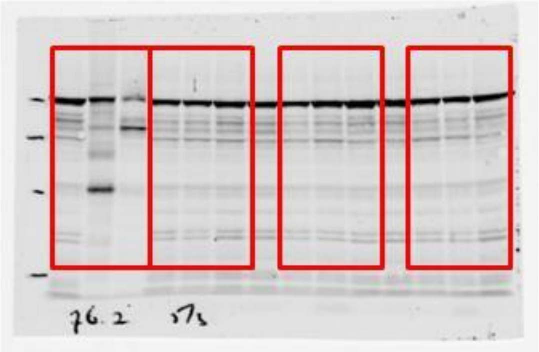

Panels E, F

Section used in Figure 6E
marked in red

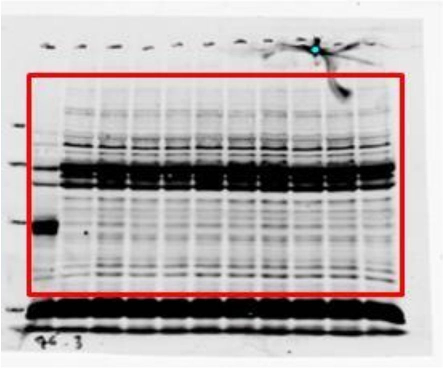

Section used in Figure 6F
marked in red

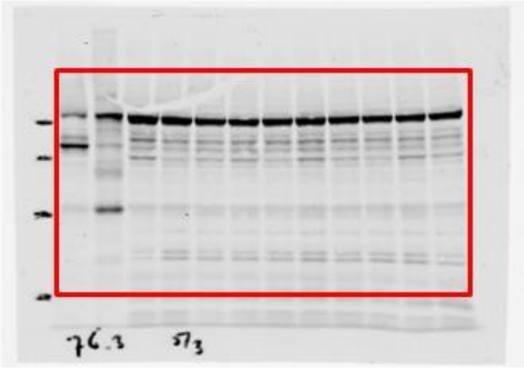

Figure 8

Panel A

Section used in Figure 8A marked in red

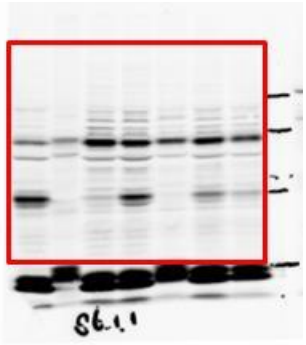

Panel B

Section used in Figure 8B marked in red

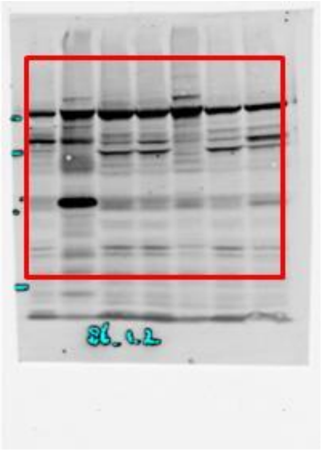

Panel C

Section used in Figure 8C marked in red

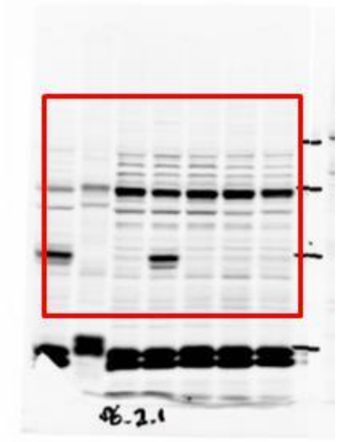

Panel D

Section used in Figure 8D
marked in red

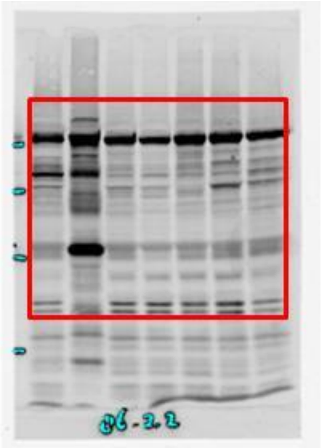

Supplementary Figure 1

Section used in Supplementary Figure 1A marked in red

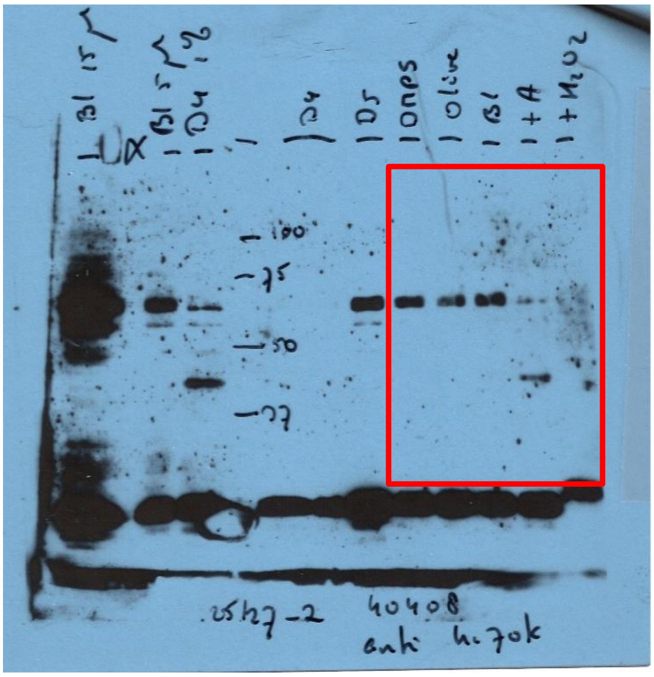

Section used in Supplementary Figure 1B marked in red

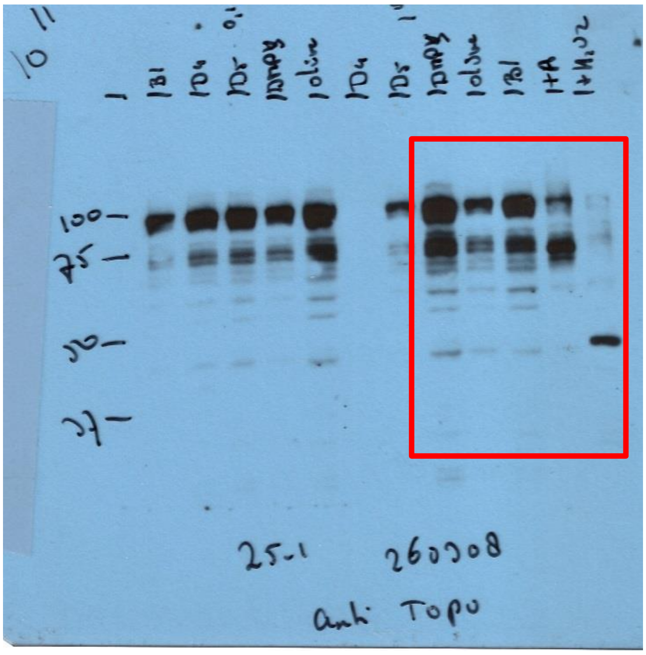

Supplement: Supplementary file 1 — Supplementary information. [file 41598_2020_66666_MOESM1_ESM.pdf]
